# Reduced Blood Pressure of CFTR-F508del Carriers Correlates with Diminished Arterial Reactivity Rather than Circulating Blood Volume in Mice

**DOI:** 10.1371/journal.pone.0096756

**Published:** 2014-05-06

**Authors:** Veronica A. Peotta, Prasad Bhandary, Ugochi Ogu, Kenneth A. Volk, Robert D. Roghair

**Affiliations:** Department of Pediatrics, University of Iowa Carver College of Medicine, Iowa City, Iowa, United States of America; Duke University Medical Center, United States of America

## Abstract

The F508del mutation of the cystic fibrosis transmembrane conductance regulator (CFTR) is the most common cause of cystic fibrosis (CF). Both CF patients and F508del carriers have decreased blood pressure. While this has been attributed to salt depletion, recent studies have shown F508del expression interferes with smooth muscle cell calcium mobilization. We tested the hypothesis that carriers of the F508del mutation have lower adult blood pressures and reduced aortic contractility without a reduction in circulating blood volume. By radiotelemetry, F508del heterozygous mice had significantly lower arterial pressures than wild-type C57BL/6 controls, with the greatest effect seen at the time of dark-to-light cycle transition (mean difference of 10 mmHg). To replicate the vascular effects of sympathetic arousal, isoproterenol and epinephrine were co-infused, and F508del mice again had significantly reduced arterial pressures. Aortas isolated from F508del heterozygous mice had significantly decreased constriction to noradrenaline (0.9±0.2 versus 2.9±0.7 mN). Inhibition of wild-type CFTR or the inositol triphosphate receptor replicated the phenotype of F508del aortas. CFTR carrier status did not alter circulating blood volume. We conclude the CFTR-F508del mutation decreases aortic contractility and lowers arterial pressures. As a cAMP-activated chloride channel that facilitates calcium mobilization, we speculate wild-type CFTR co-activation during adrenergic receptor stimulation buffers the vasodilatory response to catecholamines, and loss of this compensatory vasoconstrictor tone may contribute to the lower arterial pressures seen in heterozygote carriers of a CFTR-F508del mutation.

## Introduction

Cystic fibrosis (CF) is the most common life-limiting autosomal recessive disease [1]. It is caused by mutations of the gene encoding the cystic fibrosis transmembrane conductance regulator (CFTR), a cAMP-activated chloride channel. The predominant CFTR-F508del mutation, carried by 2–3% of individuals of European descent [2], disrupts the biosynthetic processing of CFTR such that the protein is retained in the endoplasmic reticulum and degraded [3], [4]. Individuals homozygous for the F508del mutation have classic CF-related morbidities, including pulmonary disease, gastrointestinal abnormalities, and pancreatic insufficiency. However, little is known about the direct effect of CFTR and the common CFTR mutations on cardiovascular physiology.

Patients with CF, as well as CFTR-F508del carriers, have decreased blood pressures [5]–[8]. As some have speculated [7], the F508del mutation’s evolutionary persistence may be related in part to this antihypertensive effect. Alternatively, the cardiovascular effects of the CFTR-F508del mutation could contribute to morbidity and mortality if a hypotensive phenotype correlates with altered tissue perfusion or elicits a neurohumoral response that could exacerbate the secondary pulmonary hypertension seen in cystic fibrosis patients with chronic lung disease. Intriguingly, the lower blood pressure seen in heterozygous F508del women is most dramatic in those with the highest sweat chloride levels, suggesting a potential explanatory relationship between blood pressure and fluid or electrolyte status [8]. However, studies have consistently shown a lack of hypovolemia in CF patients [6], [9], and an increasing number of investigations have identified functional CFTR expression in non-epithelial cells, including human bronchial smooth muscle cells [10]. Interestingly, airway smooth muscle cells isolated from patients with the F508del mutation have reduced histamine-induced calcium release, a phenotype replicated by pharmacologic CFTR inhibition [10]. In a complementary neonatal piglet model, some of us have likewise shown the F508del mutation reduces aortic smooth muscle cell agonist-induced calcium release and aortic tone [11].

In order to further assess the cardiovascular effects of the F508del mutation, we have turned to a mouse model. Unlike CF piglets, CF mice do not develop co-morbid lung disease and arterial pressures can be measured while the mice are housed in their home environment, thereby clarifying the direct effect of CFTR on blood pressures [12], [13]. With human data suggesting a correlation between reduced chloride gating and decreased blood pressure [8] and cell culture data suggesting the F508del mutation interferes with agonist-induced calcium mobilization [10], [11], we hypothesized that the F508del mutation reduces arterial tone and lowers arterial pressures in the absence of hypovolemia.

## Methods

### Radiotelemetry

All procedures were performed within the guidelines of the Animal Welfare Act and the National Institutes of Health *Guide for the Care and Use of Laboratory Animals* and were approved by the University of Iowa Animal Care and Use Committee (Permit Number: 1306101). CFTR-F508del mice were generated at the University of Iowa Gene Therapy Center [14]. At 4 to 5 months, wild type (WT) and F508del mice were instrumented with carotid radiotelemeters (Data Sciences International, St Paul, MN) under isoflurane anesthesia with the transmitter housed in a subcutaneous pouch. Following 7 days of recovery, blood pressures and body temperature were sampled for 10 s every 5 min during an 84 h long uninterrupted series of dark and light cycles, as previously described [15]. The arterial waveforms were utilized to derive blood pressures and pulse rate with proprietary software (Dataquest A.R.T., Data Sciences International). During the subsequent light cycle, between 0900 and 1000, the mice were awoken and arterial pressures plus body temperature were recorded at a sampling rate of 1000 Hz. Normal saline (0.9% NaCl, 10 mL/kg) was administered by intraperitoneal injection 20 min into the continuous recording and the transmitters were turned-off after 2 h. At the same time the following day, the transmitters were turned on by passing a magnet under the home cage, and the same recordings were obtained, this time with intraperitoneal administration of the beta-adrenergic receptor agonist isoproterenol (2 mg/kg) plus epinephrine (2 mg/kg) 20 min into the 2 h recording [16], [17].

### Blood Volume Determination

Jugular venous catheters were placed in an additional group of mice during isoflurane anesthesia. The following day, 500 µL of blood was obtained from a C57BL/6 donor mouse via cardiac puncture, and the harvested red blood cells (RBCs) were isolated by centrifugation. Utilizing methods analogous to those described for sheep [18], the RBCs were washed and re-suspended to a hematocrit of 25%, as measured by an automated hematology analyzer (Sysmex XE-2100, Sysmex Corp, Kobe, Japan). The washed RBCs were then biotinylated by adding 1 mg/mL Biotin-S-NHS (Thermo Scientific, Rockford, IL) and incubating at room temperature for 30 min. Donor RBCs were then washed, the RBC count determined, and 470 to 530 million biotinylated RBCs (50 µL) were transfused over 10 min via the jugular catheter. One hour after the transfusion, 40 µL of blood was sampled by cardiac puncture or from a free flowing tail vein incision. Hematocrit and mean corpuscular volume were measured with a Sysmex XE-2100. The ratio of biotinylated donor to non-biotinylated recipient RBCs was determined by flow cytometry (FACScan, Becton Dickinson, Franklin Lakes, NJ) after co-labeling all RBCs with FITC-Streptavidin. Blood volume was calculated using the following formula: (number of transfused RBC × mean corpuscular volume × 100) ÷ (ratio of biotinylated RBCs × hematocrit). To validate the technique, an additional 200 µL of blood was collected from the final four mice, and post-phlebotomy blood volumes were again measured after administering a second biotinylated RBC transfusion. Measured post-bleed blood volumes were compared to expected post-bleed blood volumes, as calculated using the following formula: (pre-bleed blood volume – phlebotomy losses) × pre-bleed hematocrit ÷ post-bleed hematocrit.

### Wire Myography

Thoracic aortas were obtained from additional WT and CFTR-F508del adult mice and 2.5 mm segments were assessed by wire myography (Danish Myotechnology), as previously described [19]. Constriction to KCl and noradrenaline (NA) were examined in the presence of buffer alone, the inositol triphosphate receptor (IP_3_R) antagonist 2-aminoethoxydiphenyl borate (2APB, 50 µM) [20], or the CFTR inhibitor 5-[(4-carboxyphenyl) methylene]-2-thioxo-3-[(3-trifluoromethyl) phenyl]-4-thiazolidinone (CFTRinh-172, 50 µM). Following a one hour equilibration at a resting tension of 5 mN, the aortic segments were constricted with cumulative concentrations of KCl (5 to 90 mM). After rinsing and re-equilibration, the vessels were constricted with cumulative concentrations of NA (10^−9^ to 10^−6^ M) to assess alpha-adrenergic receptor responsiveness (at low concentrations) and beta-adrenergic receptor responsiveness (at higher concentrations). All compounds were acquired from Sigma Chemical (St. Louis, MO).

### Statistical Analysis

All data are expressed as means ± SE. Values were considered statistically significant when p<0.05 by Student’s two-tailed *t*-test. Baseline hemodynamics, activity and temperature were analyzed by two way repeated measures ANOVA, factoring for genotype and time of day, followed by Bonferroni *post hoc* testing. Whenever a significant interaction was present between genotype and time of day, multiple comparison tests were utilized to evaluate for independent effects. The correlation between calculated and measured blood volumes was assessed by Pearson Product Moment Correlation. All analyses were performed by SigmaPlot 12.0 (Systat Software, Inc.).

## Results

### Perioperative Mortality

Despite maintenance on corn grit bedding and a high energy content diet (Teklad S-2335, 3.5 kcal/g), 7 out of 30 (23%) of the homozygous CFTRF^508del/F508del^ mice died prior to 4 months of age, and necropsy confirmed the presence of distal intestinal obstruction. Other than a heterozygous CFTRF^508del/+^ mouse that died on the same day as a CFTRF^508del/F508del^ littermate, there was no further preoperative mortality. Radiotelemeters were readily implanted in three CFTRF^508del/F508del^ mice, but all died by postoperative day 5. Given the high perioperative mortality of CFTRF^508del/F508del^ mice and our overarching interest in the phenotype of F508del carriers, our subsequent studies focused exclusively on WT and F508del heterozygous mice. For these mice, intraoperative mortality was 9.5% (1 of 20 WT, 3 of 22 F508del), and postoperative mortality was 13.2% (2 of 19 WT, 3 of 19 F508del).

### CFTR-induced Hypotension

**Figure 1:**
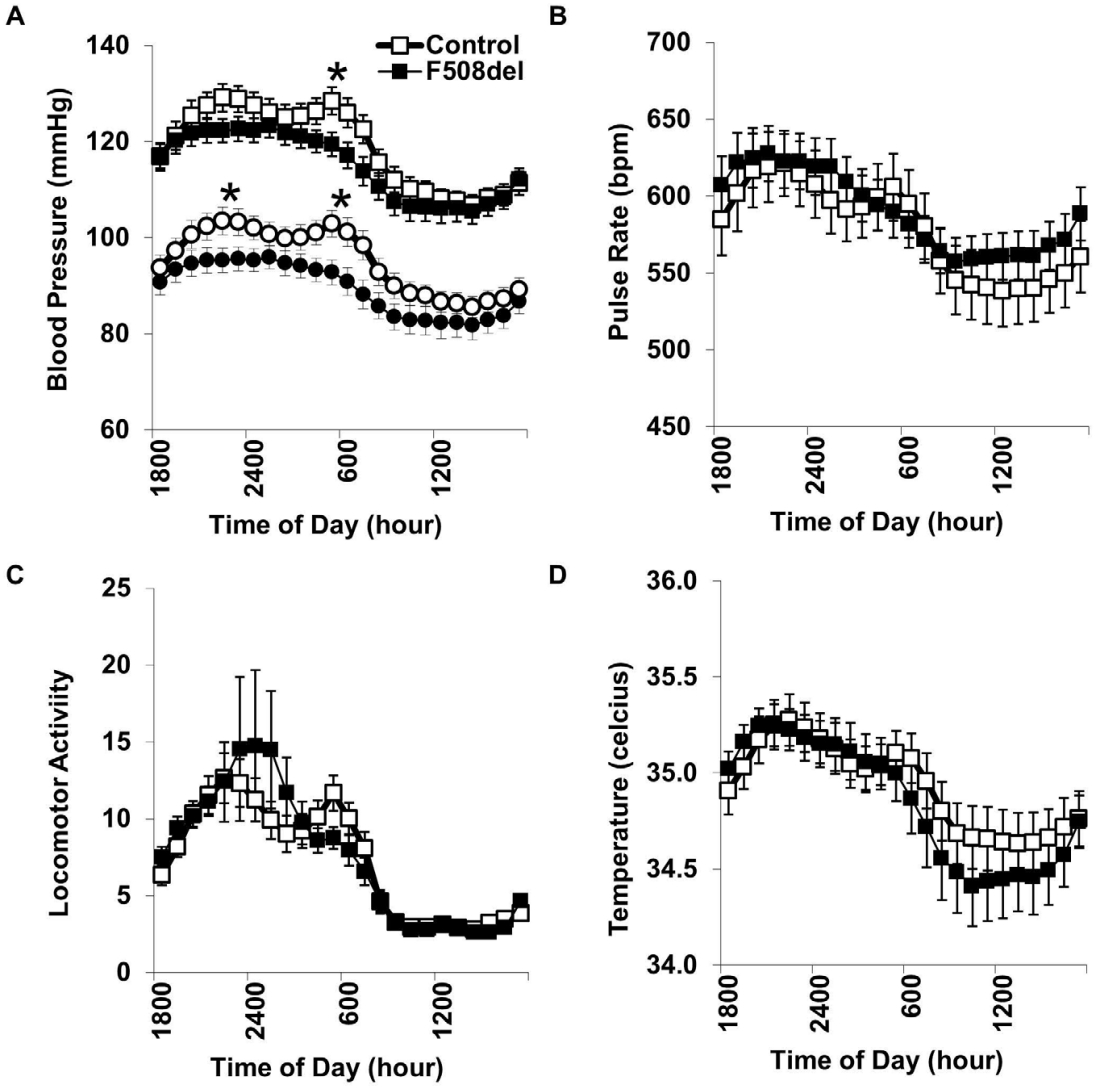
Carotid radiotelemetry. Systolic and diastolic blood pressures (squares and circles, respectively) were measured while mice were housed in their home cage during alternating cycles of darkness (1800 to 0600) and light (0600 to 1800) (A). Compared to wild-type mice (WT, open symbols), heterozygous F508del mice (solid symbols) had lower systolic and diastolic pressures at the dark-to-light transition. Pulse rate (B), locomotor activity (C), and body temperature (D) were not significantly influenced by F508del status. *P<0.05 versus WT; N = 17 WT and 16 F508del mice.

Baseline blood pressures were influenced by a significant interaction between genotype and time of day (P<0.001). By post-hoc testing, F508del heterozygous mice had significantly lower systolic and diastolic pressures, and this was the most prominent at the 0600 transition from dark to light cycles (Figure 1A). Because human studies have shown the blood pressure lowering effect of F508del status may be specific to females [8], we performed *a priori* sex-specific subgroup analysis (Table 1). Overall, female but not male blood pressures were reduced by the F508del mutation. Looking specifically at the key dark-to-light transition, dF508del mice had an overall reduction in mean arterial pressure of 9.3 mmHg (female mean difference: 9.7 mm Hg, P<0.05; male mean difference: 8.1 mm, P = 0.23). CFTR genotype did not significantly alter pulse rate, locomotor activity, or body temperature (Figure 1). Because CFTR is activated by cAMP, the key second messenger in β-adrenergic receptor activation, we speculated that the hemodynamic effects of the CFTR-F508del mutation may be further unmasked during pharmacologic β-adrenergic receptor activation.

**Table 1:** Baseline radiotelemetry.

	Female		Male	
	WT	F508del	WT	F508del
N	10	8	7	8
Systolic blood pressure (mmHg)	119±2	112±1*	119±5	118±5
Diastolic blood pressure (mmHg)	98±3	90±3	92±5	89±4
Pulse rate (bpm)	560±35	582±25	605±11	599±22
Temperature (°C)	35.2±0.1	35.3±0.1	34.6±0.2	34.4±0.1

Arterial waveform-derived systolic blood pressure, diastolic blood pressure and pulse rate were recorded, along with subcutaneous body temperature, every 5 minutes over an 84 h interval while wild-type (WT) and heterozygous F508del mice freely explored their home cage. *P<0.05 versus WT.

### Hemodynamic Responses to β-adrenergic Receptor Agonists

**Figure 2:**
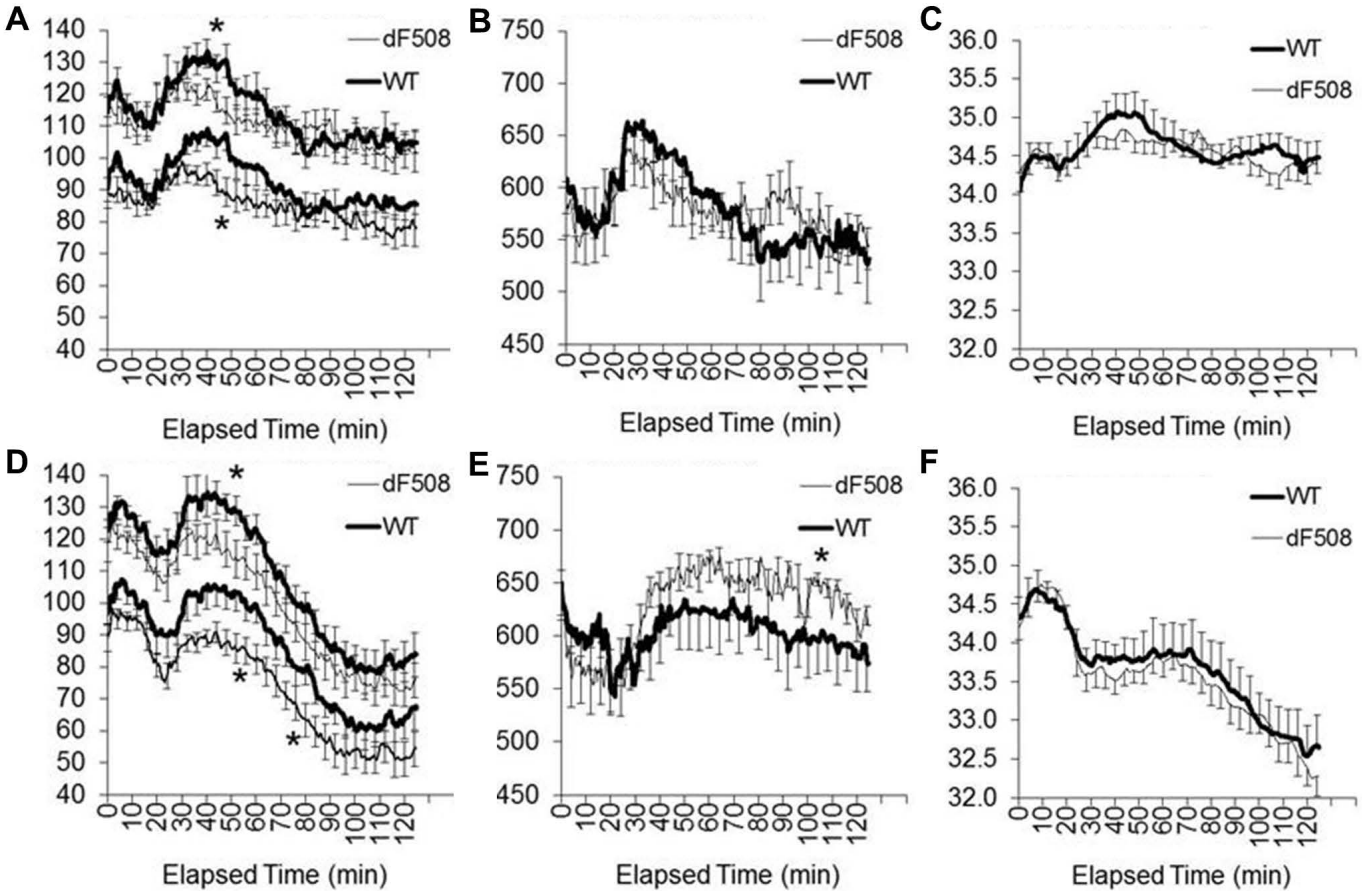
Physiological responses to β–adrenergic agonists. Twenty minutes after starting the continuous recording of arterial pressures (A), pulse rate (B) and body temperature (C), wild-type mice (WT, thick line) and heterozygous F508del mice (thin line) received normal saline (10 mL/kg i.p.). The following day, the same protocol was utilized with the injection of normal saline containing isoproterenol and epinephrine (2 mg/kg of each) (D to F). *P<0.05 versus WT; N = 14 WT and 16 F508del (injections were not given to the initial 3 WT female mice with baseline data reported in Figure 1).

Other than a brief hypertensive response to the injection, normal saline had no demonstrable effect on blood pressures, pulse rate or body temperature (Figure 2A to 2C). Consistent with the baseline data, F508del mice had lower blood pressures immediately after the stress-inducing intraperitoneal injection (Figure 2A). While the initial stress responses to co-injection of isoproterenol and epinephrine were reminiscent of the responses elicited by the normal saline injection, the second hour of recording revealed a substantial hypotensive, tachycardic and hypothermic response in both WT and F508del mice (Figure 2D to 2F). Compared to WT mice, F508del mice had exaggerated hypotensive and tachycardic responses to the co-injected β-adrenergic receptor agonists (Figure 2D and 2E, respectively). These results suggest the presence of increased β-adrenergic receptor-mediated vasodilation in F508del mice that may underlie the lower baseline pressures. Because hypovolemia enhances the hemodynamic responses to vasodilators and the presence of CFTR mutations may enhance electrolyte and water losses, circulating blood volumes were determined.

### Lack of Hypovolemia in F508del Mice

**Figure 3:**
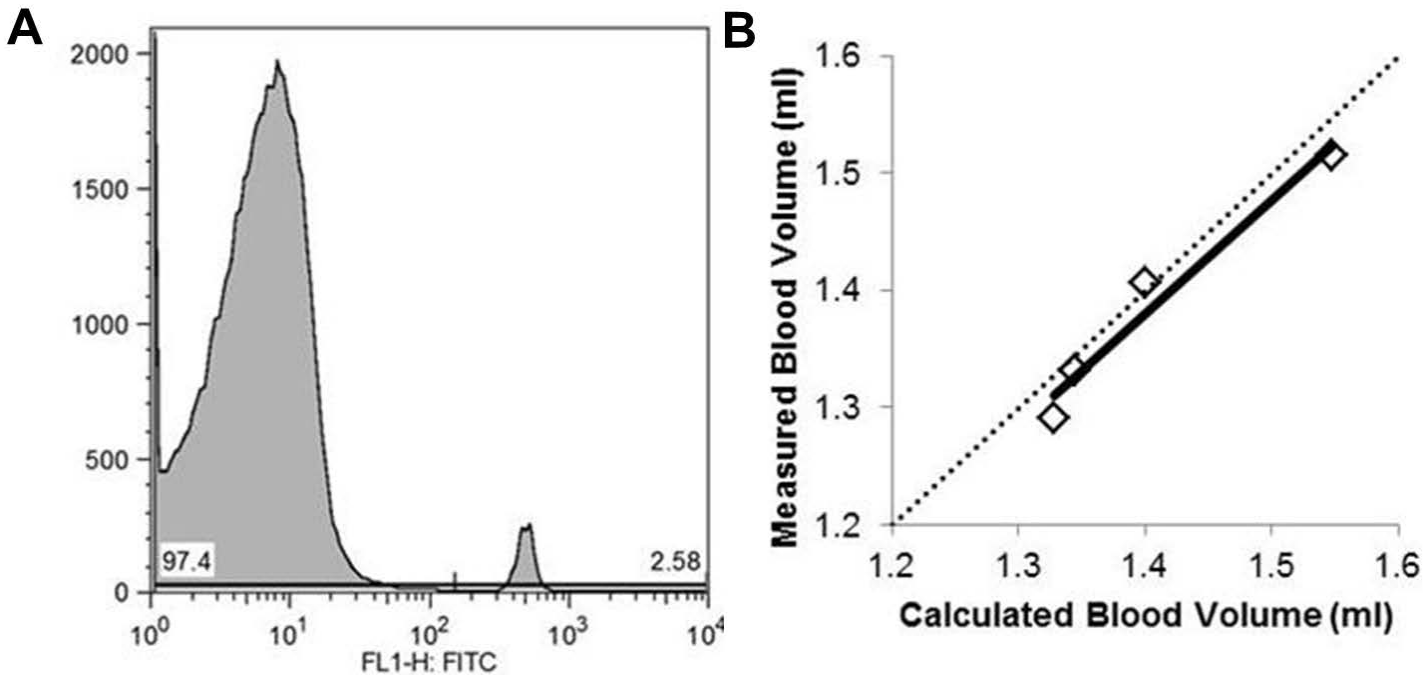
Circulating blood volume. One hour following the transfusion of biotinylated RBC, the ratio of biotinylated RBCs to native circulating RBCs was determined by flow cytometry (A, representative histogram showing 97.4% non-biotinylated RBC peak on the left and 2.6% biotinylated RBCs on the right). For 4 mice, 200 µL of blood was collected and repeat biotinylated RBC infusion was administered prior to the measurement of post-bleed blood volumes. The measured post-bleed blood volumes closely approximated the values expected based on the amount of blood removed (B, R = 0.98 and slope = 0.97 with dotted line indicating the line of identity).

The ratio of biotinylated donor and non-biotinylated RBCs was accurately quantified (Figure 3A). CFTR genotype had no significant effect on mean corpuscular volume, hematocrit or circulating blood volume (Table 2). To validate the blood volume measurements, paired samples were obtained from 4 mice before and after removal of 15–17% of the circulating blood volume. Post-bleed sampling showed a 10–14% reduction in hematocrit and a 4–10% decrease in blood volume. This is consistent with partial fluid redistribution to maintain circulating volume (Table 2). Measured and calculated blood volumes were correlated (R = 0.98, P = 0.02) as indicated by the slope of the regression line approximating the line of identity (Figure 3B). This finding bolsters confidence that if a significant difference in blood volume had been present, it would have been identified.

**Table 2:** Blood Volume.

	WT	F508del	Pre-bleed	Post-bleed
N	10	6	4	4
Mean corpuscular volume (fL)	48.6±0.2	46.9±0.5	47.8±0.7	47.6±0.8
Hematocrit (%)	44±2	39±4	49±1	43±1*
Blood Volume (ml/kg)	58±3	64±2	64±3	60±4*

One hour after infusion of biotinylated RBCs, mean corpuscular volume and hematocrit were measured along with flow cytometric determination of the percentage of donor and recipient RBCs. These values were utilized in calculating the circulating blood volume. In 4 mice (3 WT and 1 F508del), an additional 200 µl of blood was collected and a second biotinylated RBC transfusion administered prior to the measurement of post-bleed values. *P<0.05 versus pre-bleed values by paired t-test.

### CFTR-dependent Aortic Reactivity

**Figure 4:**
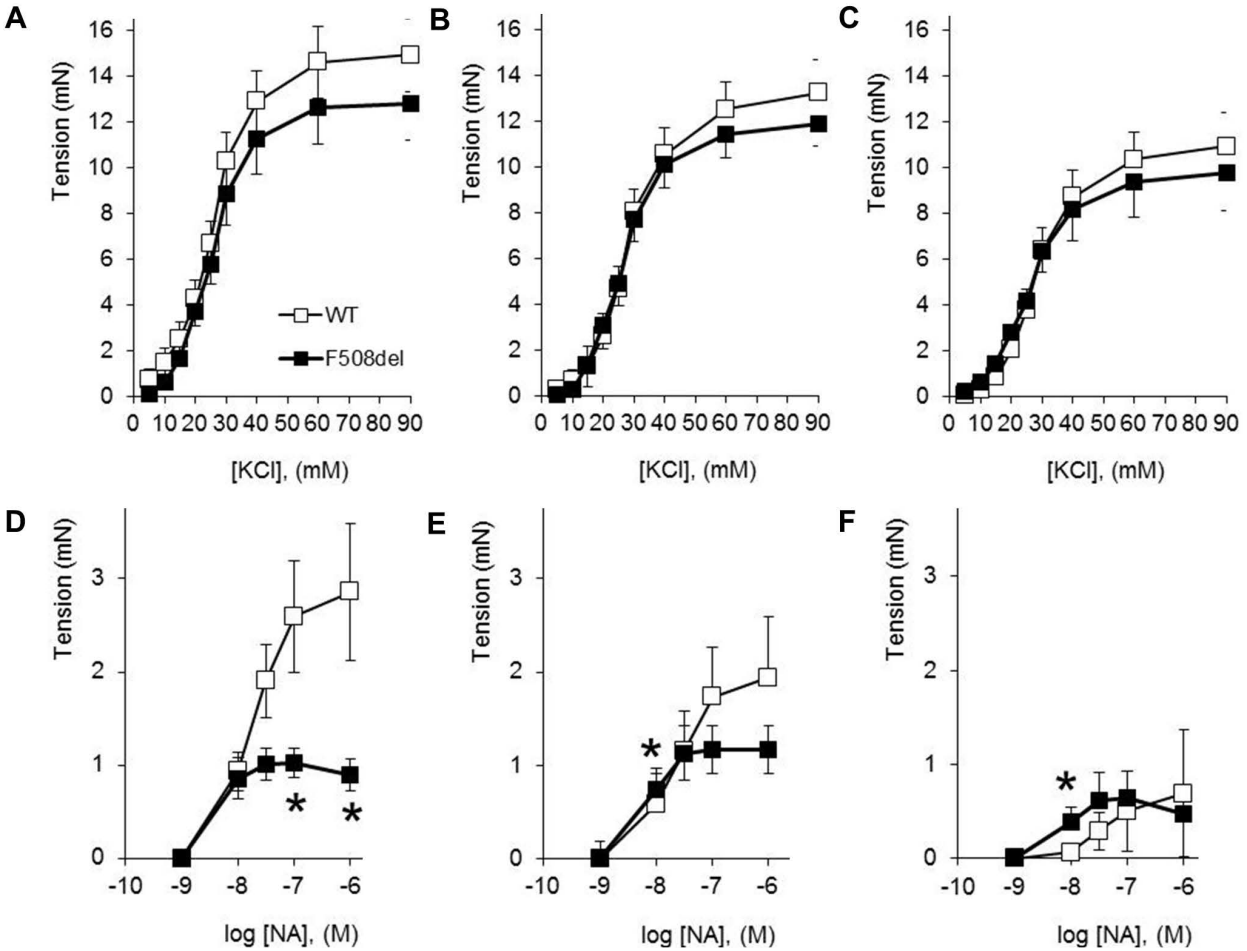
Aortic myography. Wild type (WT, open symbols, N = 9) and heterozygous F508del (closed symbols, N = 8) aortic constriction to KCl was assessed in the presence of buffer alone (A), CFTR inhibition (B, CFTRinh-172), or IP_3_R antagonism (C, 2-APB). After rinsing the arterial segments, the responses were then assessed to cumulative concentrations of noradrenaline (NA). In buffer alone, F508del aorta had attenuated constriction to NA (D). This was blocked by preincubation of the aortic segments in either CFTRinh-172 (E), or 2-APB (F). *P<0.05 versus WT.

Without evidence that the hypotensive phenotype of F508del mice is a consequence of hypovolemia, we went on to assess the effects of the F508del mutation on isolated vessel reactivity. Aortic constriction to KCl was not significantly altered by CFTR mutation status (Figure 4A). CFTRinh-172 significantly decreased the response of WT but not F508del aorta to 10–20 mM KCl (Figure 4B; 38+/−12% versus 14+/−18% decrease in constriction to 15 mM KCl, respectively), while the IP_3_R antagonist 2APB significantly decreased the response of both WT and F508del aorta to >15 mM KCl (Figure 4C). Compared to WT aorta, aorta from F508del heterozygous mice had decreased constriction to the highest concentrations of NA (Figure 4D). Although preincubation with CFTRinh-172 did not significantly decrease NA-evoked constriction of aorta from WT or F508del mice (Figure 4E), IP_3_R inhibition with 2APB decreased the NA responsiveness of WT more than F508del aorta (73+/−7% versus 3+/−59% decrease in constriction to 10^−7 ^M NA, respectively), thereby replicating the vascular effects of the F508del mutation (Figure 4F).

## Discussion

While the effects of CFTR expression on epithelial tissue are well described [21], [22], the effects of CFTR on muscle tissue are relatively undefined. Both human and murine studies have shown skeletal muscle dysfunction may be intimately linked with the respiratory insufficiency and exercise intolerance that contribute to the morbidity of patients with F508del mutations [23], [24]. Likewise, both human and murine studies suggest CFTR mutations directly interfere with cardiac myocyte function [25]–[27]. Utilizing a neonatal piglet CF model, we recently replicated human data showing decreased calcium transients in F508del expressing smooth muscle cells and further correlated this with impaired aortic constriction [11]. In that study, F508del heterozygous aortic smooth muscle cells had a phenotype intermediate between that of the WT and CF cells [11]. The present studies investigated whether a single copy of the F508del mutation is sufficient to reduce arterial pressure and arterial contractility.

As reported in human carriers, F508del heterozygous mice had significantly decreased baseline arterial pressures. In both cases, the phenotype is stronger in females than males. In the human studies, women with the lowest blood pressures tended to have the lowest level of CFTR activity, as measured by sweat chloride levels [8]. Similarly, the hypotensive effect of the mutation in mice was most pronounced during the transition between dark and light cycles, a time of heightened arousal associated with increased sympathetic tone and potentially increased CFTR activation [28]. In F508del male mice, a hypotensive phenotype was elicited during the administration of beta-adrenergic receptor agonists, compounds that increase cAMP levels and, at least theoretically, WT CFTR activity, thereby magnifying the phenotypic effect of endogenous CFTR expression. Among the intracellular processes that may contribute to the CFTR modulation of β-adrenergic signaling, there is experimental support for CFTR-mediated membrane depolarization leading to enhanced flux through voltage-dependent calcium channels [27].

To begin testing the hypothesis that cAMP-mediated WT CFTR co-activation facilitates myocyte contraction as a buffer against β-adrenergic receptor-mediated vasodilation, the reactivity of isolated aortic segments was assessed. There were no significant differences between WT and F508del aortic reactivity to depolarizing concentrations of KCl, possibly reflecting a low level of basal CFTR activation. Nevertheless, CFTR inhibition did significantly reduce the constriction of WT aorta to KCl. This contrasts with studies in epithelial cells where CFTR activation antagonizes IP_3_-mediated smooth muscle cell constriction and promotes vasodilation, possibly by hyperpolarizing the endoplasmic reticulum membrane [21], [22]. This dichotomy may originate from the differing resting membrane potentials and intracellular chloride content of the 2 cells types. As reviewed by Matchkov and colleagues, vascular smooth muscle cells have a relatively depolarized resting potential and actively accumulate chloride, allowing for chloride efflux-induced membrane depolarization [29]. Because it is difficult to achieve consistent sub-maximal aortic preconstriction prior to the addition of β-adrenergic receptor-specific vasodilators, we instead chose to assess aortic responsiveness to NA, a catecholamine with mixed α- and β-adrenergic receptor activity.

Unlike the progressive vasoconstriction seen from WT aorta, the aorta from F508del mice had significantly diminished arterial tone at the highest NA concentrations. While pharmacologic CFTR inhibition of WT responsiveness largely recapitulated the F508del phenotype, it is possible the limiting effects of the F508del mutation on vasoconstriction may extend beyond the channels chloride gating properties. In particular, the expression of CFTR-F508del may elicit an unfolded protein response that could alter endoplasmic reticulum structure and the activity of other proteins that are targets of the endoplasmic reticulum stress response, including the IP_3_ receptor [30], [31]. The IP_3_ receptor is intimately involved in the regulation of aortic reactivity, as demonstrated by the inhibitory effects of 2APB in the current study. Furthermore, the induction of an endoplasmic reticulum stress response to CFTR-F508del expression has been shown to transcriptionally repress WT CFTR [32]. A reduction in chloride gating, interference with IP_3_ receptor expression and/or a decrease in expression of the WT CFTR allele could contribute to the cardiovascular phenotypes of mice expressing but a single copy of the F508del mutation.

The circulating blood volume data complemented the myography results. Our results are comparable to those reported for wild-type mice using an alternative radiolabelled RBC method [33]. While no significant differences in circulating blood volume were seen in F508del mice, it is notable that F508del mice tended to have an increase, rather than the theoretical decrease in blood volume. This is consistent with human data showing an increase in total body water among CF patients with congestive heart failure and even relatively healthy adolescents with F508del mutations [6], [9]. The etiology of this relative hypervolemia does not appear to correlate with dietary sodium intake, but could be influenced by renin-angiotensin system activation [6].

Our studies are limited by the lack of hemodynamic measurements on CFTR-F508del homozygous mice. As previously reported, CF mice have increased mortality, primarily attributed to a distal intestinal obstruction syndrome [14]. Further investigations are needed to reconcile the demonstrated reduction in vascular reactivity with clinical data showing increased aortic stiffness and impaired flow mediated vasodilation in F508del homozygous patients [34], [35]. In both studies, the increase in aortic stiffness or decrease in vasodilation correlated with inflammatory markers and the severity of lung disease, and it is thus difficult to extrapolate from homozygous to heterozygous individuals. As an endothelium-dependent process, the reduction in flow-mediated dilation suggests the need to further assess the effects of endothelial cell-restricted CFTR-F508del expression [36]. Likewise, because adult blood pressures are influenced by factors other than arterial tone, further investigations centered on vascular smooth muscle-specific F508del expression are needed to clarify the causal relationship between CFTR mutations and the observed reductions in arterial pressure. While CFTR null mice might serve well as a genetic background to introduce vascular-specific F508del expression, studies on murine aortic smooth muscle cells and cardiomyocytes show the loss of CFTR in these cells strongly upregulates alternative chloride channels, as well as voltage-dependent calcium channels [27], [37].

In an autosomal dominant inheritance pattern, mice with an F508del mutation have decreased aortic responsiveness and lower arterial blood pressures. The pathophysiological implications of these findings are unknown. While the hypotensive phenotype may be considered an advantage, if it is a manifestation of dysregulated vascular tone, the exaggerated hypotensive response to adrenergic stimulation may interfere with the regulation of blood flow at times of sympathetic activation. Reassuringly, young adults with relatively mild cystic fibrosis do not appear to have basal or exercise-induced sympathetic over-activation, as measured by circulating norepinephrine levels [5]. With studies showing increased urinary and adrenal catecholamines in patients with late-stage cystic fibrosis [38], further studies are needed to track the state of sympathetic activation, vascular tone, blood pressure and tissue perfusion during the evolution of cystic fibrosis-induced lung disease. There are active clinical trials of small molecules capable of stabilizing CFTR-F508del and enhancing plasma membrane CFTR expression [1], [39]. As these systemically administered medications are increasingly used to modify CFTR expression or activation, our understanding of the physiological effects of extra-pulmonary CFTR expression must keep pace.
